# Providing manualized individual trauma-focused CBT to unaccompanied refugee minors with uncertain residence status: a pilot study

**DOI:** 10.1186/s13034-019-0282-3

**Published:** 2019-05-17

**Authors:** Johanna Unterhitzenberger, Svenja Wintersohl, Margret Lang, Julia König, Rita Rosner

**Affiliations:** Department of Psychology, Catholic University Eichstätt-Ingolstadt, Ostenstrasse 25, 85072 Eichstätt, Germany

**Keywords:** Treatment, Refugee, Asylum seeker, Adolescents, PTSD, Trauma, TF-CBT

## Abstract

**Background:**

Unaccompanied refugee minors (URMs) seeking asylum show high rates of posttraumatic stress disorder (PTSD), depression and anxiety. In addition, they experience post-migration stressors like an uncertain residence status. Therefore, psychotherapeutic interventions for URMs are urgently needed but have scarcely been investigated up to now. This study aimed to examine manualized individual trauma-focused cognitive behavioural therapy (TF-CBT) for URMs with PTSD involving their professional caregivers (i.e. social workers in child and adolescent welfare facilities).

**Methods:**

We conducted an uncontrolled pilot study with three follow-up assessments (post-intervention, 6 weeks, and 6 months). Participants who met the PTSD diagnostic criteria were treated in a university psychotherapeutic outpatient clinic in Germany with a mean of 15 sessions of TF-CBT. All participants (n = 26) were male UM (M_age_ = 17.1, SD = 1.0), predominately from Afghanistan (n = 19, 73.1%) and did not have a residence permit. The sample was severely traumatized according to the number of traumatic event types reported (M = 11.3, SD = 2.8). The primary outcome was PTSD measured with the Child and Adolescent Trauma Screen (CATS) and the Diagnostic Interview for Mental Disorders in Childhood and Adolescence (Kinder-DIPS). Secondary outcomes were depression, behavioural and somatic symptoms. All but the somatic symptoms were assessed in both self-report and proxy report.

**Results:**

At post-intervention the completer sample (n = 19) showed significantly decreased PTSD symptoms, *F*(1, 18) = 11.41, *p *= .003, with a large effect size (d = 1.08). Improvements remained stable after 6 weeks and 6 months. In addition to PTSD symptoms, their caregivers reported significantly decreased depressive and behavioural symptoms in participants. According to the clinical interview, 84% of PTSD cases recovered after TF-CBT treatment. After 6 months, youths whose asylum request had been rejected showed increased PTSD symptoms according to individual trajectories in the Kinder-DIPS. The effect was, however, non-significant.

**Conclusions:**

Intervention studies are feasible with URMs. This pilot study presents preliminary evidence for the efficacy of an evidence-based intervention like TF-CBT in reducing PTSD symptoms in URMs. Stressors related to asylum proceedings after the end of therapy have the potential to negatively influence psychotherapy outcomes.

## Background

Research conducted over the last 10 years throughout Europe suggests that unaccompanied refugee minors^1^ (URMs) who have relocated to European countries have experienced a high number of pre-, peri-, and post-migration traumatic events [1–3] and face various mental health problems in exile, especially posttraumatic stress disorder (PTSD), depression and anxiety [2, 4–6]. Given their diverse cultural backgrounds, psychological symptoms in young refugees are often linked to a higher degree of somatic problems [3]. In addition, they suffer from post-migration stressors like an uncertain residence status and isolation [7, 8]. Suicidal and self-harming behaviour seem to be more common in URMs than in non-refugee youths [9]. The mental health trajectories of URMs in Norway showed that the psychological distress reported immediately after arrival in the country remained stable over 21 resp. 26 months [10, 11]. URMs who were given a residence permit did not improve on mental health scales, and those who were refused asylum reported further increased distress [10]. Hence, mental health support and, more particularly, interventions for PTSD are very much in demand. This demand increased further after the so-called refugee crisis starting in 2015 which has impacted not only European countries but also the USA. However, URMs do not have sufficient access to psychiatric or psychotherapeutic care [9, 10, 12]. There are several reasons for this. Young refugees often have limited knowledge about the healthcare system and how to access it. They fear stigmatization and may have different concepts of mental health problems and their treatment. In addition, the host country often limits access to the healthcare system. An example, URMs are often not allowed to have health insurance. Furthermore, bilingual therapists and translators are few and far between, especially in rural areas. Many therapists avoid working with URMs due to a lack of knowledge about the administrative or intercultural characteristics of working with them.

Trauma-focused cognitive behavioural therapy (TF-CBT) [13] is an evidence-based individual psychotherapy for children and adolescents suffering from PTSD. At present, more than 20 randomized controlled trials (RCTs) support its efficacy and effectiveness and international guidelines recommend it as first-line treatment for traumatized youths [14, 15]. Its effects are stable [16] and it has been shown to also decrease comorbid symptoms of depression and anxiety [17]. Findings for cultural sensitivity of TF-CBT [18, 19] and a recent case series with URMs [20] support its feasibility with young refugees. Even if URMs are in transition to adulthood, TF-CBT offers some promising treatment characteristics for this group. As there is a high level of caregiver involvement, TF-CBT is specifically suited to improving social networks and support—resources that URMs often lack [21]. It has been studied with participants from ages three to 18 [22, 23]. Consequently, the level of language requirements can be adjusted to the individual patient. Limited language skills or the involvement of translators are not supposed to be barriers to TF-CBT. So far, there has been a lack of treatment studies focusing on URMs with PTSD, especially regarding RCTs and follow-up assessments [24]. The reasons for the weaknesses in treatment study quality with URMs could be their precarious residence status, pending asylum hearings and relocations to other accommodation or regions. Researchers and therapists do not, therefore, know how long a patient will actually be available for therapy and assessment. Furthermore, a wait list control group could be deemed to be unethical as participants could face deportation while waiting for treatment. Ehntholt, Smith, and Yule [25] for instance, reported a 50% attrition rate at follow-up, despite a relatively short follow-up period of 2 months, in their CBT group intervention for refugee children (23% URMs). Moreover, participants showed increased symptom severity at follow-up compared to post-treatment which was discussed as possibly being linked to a recent instability in the children’s home countries at that time. In summary, research shows that URMs constitute a group with an urgent and largely unmet need for treatment, that this group can probably be successfully treated with existing treatments for PTSD, and that research with this group faces several obstacles. A pilot study is, therefore, needed to document these obstacles and ways of overcoming them, and to prepare the procedures for a full-scale RCT with this target group.

In this study we investigated the efficacy of individual TF-CBT for a sample of URMs who had been diagnosed with PTSD, and—for the first time—the long-term stability of the effects, while documenting asylum procedures during psychotherapy and follow-up in a pilot study. We hypothesized (1) a significant reduction in PTSD diagnoses and symptoms (primary outcome), (2) significant reductions in comorbid depressive, behaviour and somatic symptoms (secondary outcome) after TF-CBT treatment, and (3) stability of symptom reductions in primary and secondary outcomes in follow-up assessments. We expected to find those reductions in both self-reports and caregiver reports. Furthermore, we aimed to examine whether adverse events, such as asylum refusal, have the potential to influence PTSD symptoms in a negative way even after receiving psychotherapy.

## Methods

### Participants and procedure

All participants were treated at the psychotherapeutic outpatient clinic of the Catholic University Eichstätt-Ingolstadt. The inclusion criteria were: (1) arrived in Germany unaccompanied and under the age of 18, (2) current age no older than 21, (3) PTSD diagnosis according to the Diagnostic and Statistical Manual of Mental Disorders, 5th edition (DSM-5) [26], (4) living in a facility run by the German child and adolescent welfare (CAW) agency, (5) stability of living situation (at least 4 weeks in the current group home), and (6) availability of a caregiver to take part in assessment and psychotherapy. Youths were excluded from study participation in the case of (1) acute suicidality or risk of harm to others, (2) acute life-threatening self-harm, (3) bipolar disorder, (4) psychotic disorder, and (5) acute substance abuse. Caregivers who accompanied participants to treatment were professionals (e.g. social workers), who worked in the CAW facilities where participants lived. They had to have known the patient for at least 4 weeks and the patient had to see them as trustworthy. To ensure that this was the case, we added the inclusion criteria 4, 5, and 6. Furthermore, as PTSD treatments are known to work best in persons with a PTSD diagnosis, we decided to include only URMs with a full-blown PTSD. The reason we included participants up to the age of 21 is that, in the German health care system, child and adolescent psychotherapists are allowed to treat young adults up to the age of 21.

**Fig. 1:**
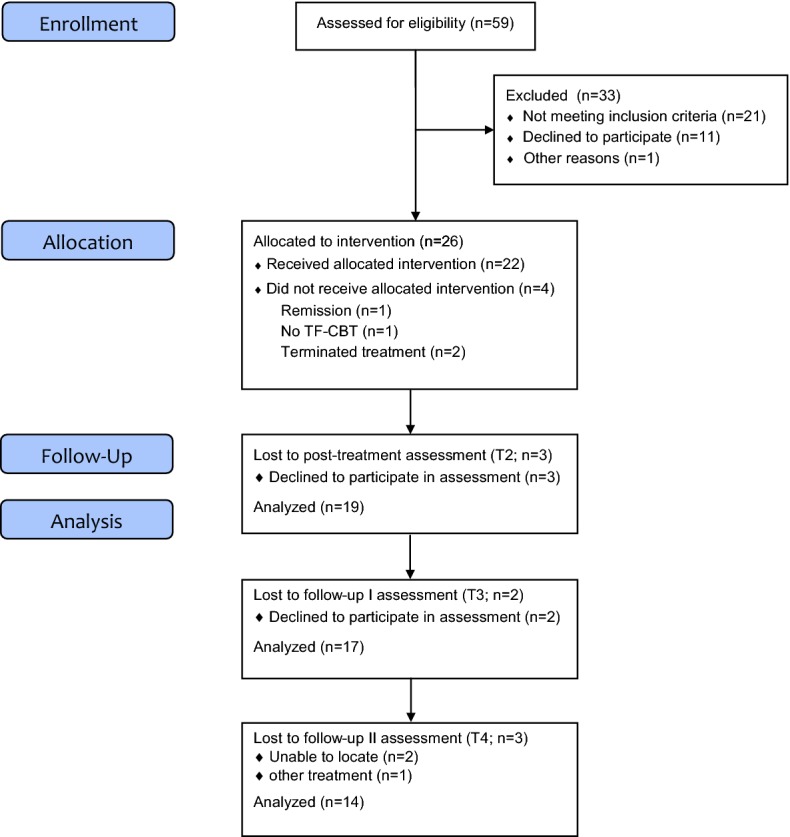
Participant flow

Participants were generally referred by staff from the CAW facilities where they lived. Youths and their respective caregivers were invited to an initial meeting with the first author, where the treatment and the study were explained to them and a first screening took place. Interpreters were on hand to assist during the appointments whenever necessary. If screened positively, the next step was the pretreatment assessment (T1). If the inclusion criteria were confirmed, the youth was offered the intervention (Fig. 1). We conducted assessments 1 week (T2), 6 weeks (T3) and 6 months (T4) after the end of treatment. Participants received vouchers as an incentive for T3 (10€) and T4 (15€) assessments.

The study was conducted between March 2015 and July 2017 and was approved by the ethical review board of the Catholic University Eichstätt-Ingolstadt. Informed consent was given by the youth, the caregiver, and—in the case of minors—by their legal guardian.

### Measures taken to reduce attrition

As shown above, URMs constitute a difficult target group for methodologically sound intervention research. To make it easier for URMs to engage and stay in treatment, we involved trusted caregivers from the initial interview onward, and made sure that interpreters were available where needed and seen as trustworthy by the participants. This also involved the participants being able to choose the interpreter’s gender. Furthermore, we took great care to educate participants about psychotherapy in general and about confidentiality in particular (information sheets were prepared in several languages to this end and handed out at the initial meeting). In addition, participants were given a 10€ (T3) or 15€ (T4) voucher as an incentive to participate in follow-up assessments.

We regularly asked for informal feedback on assessment and therapy. Formal feedback involved participants’ rating of assessment-related experiences (RARE; Rimane & Vogel, unpublished test) after baseline diagnostics which led for example to a reduction in the number of questionnaires. Please refer to the Measures section for further information.

### Treatment

TF-CBT consists of nine modules that can be illustrated within the acronym PRACTICE [13]. The first five components, psychoeducation and parenting skills, relaxation, affective modulation, and cognitive processing, are trauma-focused stabilisation skills to prepare patients for describing their personal trauma experiences and to cope with their symptoms related to these experiences. This is followed by the trauma narrative and cognitive processing II (in sensu exposure), and in vivo exposure work. After the narrative has been processed, there is a conjoint child/caregiver session and a module focusing on enhancing safety and future skills in order to integrate the traumatic events into the child’s life [13]. TF-CBT is trauma-focused; it emphasizes the need for caregiver involvement and skills, and works with graduated exposure from the very beginning. The TF-CBT manual suggests a 1:1 ratio of child and caregiver sessions. However, this can be modified according to the patient’s age. In this study, the level of caregiver involvement was flexible and modified to the individual participant’s age and need. Participants received a mean of 15 sessions of TF-CBT (100 min each). On average the therapists saw the caregiver in 8 sessions (53.3% of participants’ sessions). In all but one treatment case there was a conjoint session with patient and caregiver. Treatment cases were conducted by eight therapists (one male) who were licensed in Germany or undergoing training to become licensed psychotherapists. All therapists completed the TF-CBT online training in English or German and attended a 2-day TF-CBT training run by a licensed TF-CBT trainer (RR). Therapists underwent in-house supervision biweekly (RR). In addition, they had case consultation calls with one of the treatment developers, Anthony Mannarino, once a month. If therapists missed more than 30% of supervision sessions and/or failed to record any treatment session on videotape, the case was excluded from the trial as adherence to TF-CBT could not be verified (“no TF-CBT”, Fig. 1). Treatment fidelity was checked by two independent raters who randomly viewed three videotaped sessions of each participant. Therapists completed treatment checklists after each session as a self-report measure of adherence and to document changes in the manual course (mean adherence was rated as 82% in URM and 62% in caregiver sessions). An interpreter was present in 55% of treatment cases.

In terms of TF-CBT components and dosage, we carefully documented modifications with the help of treatment checklists and made the following observations. In addition to psychoeducation on PTSD and traumatic events, therapists provided psychoeducation on psychotherapy, working with translators, and a focus on the obligation to preserve confidentiality. In some cases the affective modulation played a major role in the first phase of treatment. For instance, skills had to be introduced already in the first session or more sessions were needed to practice naming and recognizing feelings. The trauma narrative was developed over several sessions. It always started with a time line to structure the traumatic experiences and identify the index event(s). Many URMs had lost family members or had missing persons in their families. Therefore, grief-specific components of TF-CBT [13] were added after the trauma narrative if necessary. In addition, we used grief specific material for the loss of homeland to address homesickness (e.g. “What I miss and what I don’t miss about Afghanistan”) and to resolve ambivalent feelings. All participants worked with their therapists on “Strategies for a good future” in the last treatment phase. This included helpful strategies learnt in treatment, helpful persons or sentences. In some cases, an emergency safety plan was developed and practiced in the event of a refusal of asylum (i.e. who to call, what actions to take). The involvement of translators did not present any issues in implementing TF-CBT.

### Measures

#### Primary outcomes

The Diagnostic Interview for Mental Disorders in Childhood and Adolescence (Kinder-DIPS) in German [27] includes a child and caregiver interview. It is deemed to be a valid structured interview for mental disorders in children aged 6 to 19, with good psychometric properties of the German version [28]. The Kinder-DIPS was used to determine PTSD diagnostic status according to the DSM-5 [26] and comorbid diagnoses. We assessed current diagnoses only.

We used the German version of the Child and Adolescent Trauma Screen (CATS) [29] in the self-reports and caregiver reports. CATS is a screening questionnaire for exposure to potentially traumatic events and PTSD symptoms according to DSM-5. The reliability of the German version is good to excellent [29] and Cronbach’s alpha in this study was .82 (self-report) and .74 (caregiver report). The cut-off for clinically relevant symptoms is ≥ 21 (range of scores 0–60). In our study 4 events were added to the original 15-item event list, that proved to be relevant for URMs: “several days without enough water or food”, “dangerous transport/travel”, “kidnapping, imprisonment, deportation”, and “laid (forced to or voluntary) violent hands on someone”.

#### Secondary outcomes

The Mood and Feelings Questionnaire (MFQ) [30] is a self-report and caregiver report questionnaire to assess depressive symptoms. We used the German short version with 13 items that measures symptoms on a 3-point Likert scale. Cronbach’s alpha in our study was .88 (self-report) and .77 (caregiver-report). The cut-off for clinical relevant symptoms was ≥ 12 (range of scores 0–26).

By using the Strengths and Difficulties Questionnaire (SDQ) [31] in the self-reports and caregiver reports, we measured 25 behavioural attributes divided into five subscales: emotional symptoms, conduct problems, inattention-hyperactivity, peer problems and pro-social behaviour. The total difficulties score comprises all but the last scale. The SDQ uses a three-point Likert scale. In a British sample reliability was good [32]. In our sample where we used the German version of the SDQ, Cronbach’s alpha was .74 (self-report and proxy report).

The Patient Health Questionnaire Physical Symptoms (PHQ-15) [33], German version, was used to screen for physical symptoms. As our sample was all-male, we omitted the item on menstrual cramps. The total score ranges from 0 to 30. In this study Cronbach’s alpha was .74.

The Kinder-DIPS was administered by trained bachelor or master level psychologists for both youth and caregiver. Interpreters supported assessments when needed. The CATS, MFQ and SDQ were completed by patient and caregiver on tablet devices. Raters were on hand to assist both participants in case items were difficult to understand and interpreters to make sure all wording was sufficiently understood and could be translated correctly. Therapists did not take part in any of the assessments to avoid biased results. As there was no control group, we could not guarantee full blinding of raters. However, we tried to use different raters for each assessment (T1, T2, T3, T4) whenever possible to prevent them from drawing conclusions about the participant’s treatment status within the study. Originally, we were going to include the Adolescent Dissociative Experiences Scale (A-DES) [34] and the Screen for Child Anxiety Related Emotional Disorders (SCARED) [35]. However, we dropped these measures due to insufficient validity and reliability, participants reporting difficulties in understanding the items and inappropriate questions (e.g. separation anxiety regarding parents for separated youths). Furthermore, participants gave the feedback that the assessment sessions lasted too long and this was confirmed by raters. Suicidality was assessed after every assessment by a licensed psychotherapist (JU). During treatment, the respective therapist was responsible for screening for suicidality in his/her patient after every session.

### Data analysis

We used SPSS statistics version 25 for Windows for all analyses. We report descriptive data for demographic and baseline data and the number of reported traumatic events. The primary outcome (CATS) was analysed using multivariate analyses of variance (MANOVAs, for self-report and proxy report) for the comparisons T1–T2, T1–T3 and T1–T4 separately due to differing sample sizes. We tested changes in PTSD diagnostic status (Kinder-DIPS) using the McNemar test for dependent samples. We used a repeated measures MANOVA (without T4 data due to missing data) and post hoc t-tests to examine symptom reduction regarding secondary outcomes. Given the pilot nature of this study we conducted all analyses with available samples at each time point (“completer sample”) and we reported the sample size at each time point. Furthermore, we used an uncorrected significance level of .05 (2-tailed) for all analyses due to the exploratory nature of the hypotheses. Cohen’s effect size d was calculated for within group comparisons. On the individual level clinically meaningful symptom reduction for the primary outcome (CATS) was assessed using the reliable change index (RCI) [36]. This resulted in changes > 13 points being considered as reliable changes.

## Results

### Sample at baseline

As illustrated in Fig. 1, the sample consisted of N = 26 youth (100% male) receiving TF-CBT. The mean age was M = 17.1 (SD = 1.0) with an age range of 15–19 years (Table 1). Treatment was completed by 22 participants, i.e. the drop-out rate was 15.4%. The reasons for drop-out were spontaneous remission in one case and one case was considered as “no TF-CBT” as the therapist did not participate in supervision. In two cases, after the patient repeatedly cancelled sessions, the therapist and the patient agreed to terminate treatment altogether. A further three participants were not available for post-assessments. The majority of URMs came from Afghanistan and most had lost at least one parent to death. One-third had no contact to any family members at all. The mean number of types of traumatic events was very high (M = 11.3, SD = 2.8) and the events reported most frequently were: dangerous transport (n = 25, 96.2%), lack of water and/or food (n = 25, 96.2%), experience of war (n = 24, 92.3%), sudden death of a loved one (n = 21, 80.8%), witness of violence outside family (n = 21, 80.8%), experience of violence outside family (n = 20, 76.9%), imprisonment (n = 20, 76.9%), witness of violent attack with weapon (n = 19, 73.1%) and witness of violence inside family (n = 19, 73.1%). One-third reported a suicide attempt in the past and two-thirds suicidal thoughts at least once before or at the present time. Comorbid disorders were present in 76.9% of cases with affective disorders being diagnosed most frequently.

**Table 1:** Demographic and baseline characteristics of study participants

Variable (n = 26)	M (SD), range
Age	17.1 (1.0), 15–19
Time in Germany (months)	9.8 (3.9), 4.5–21
Years of education (n = 24)	5.6 (3.7), 1–12
Number of traumatic event types	11.3 (2.8), 6–17

Variable (n = 26)	n (%)
Nationality	
Afghanistan	19 (73.1)
Eritrea, Gambia, Iran, Sierra Leone, Somalia, Sudan, Syria	Each 1 (3.8)
Religion	
Islam	23 (88.5)
Christianity	3 (11.5)
Loss of one parent	14 (53.8)
Loss of both parents	7 (26.9)
No contact to any family	8 (30.8)
Self-harm lifetime	17 (65.4)
Suicidal thoughts	16 (61.5)
Attempted suicide pre-enrolment	7 (26.9)
Comorbidity	20 (76.9)
Major depression	12 (46.2)
Dysthymia	4 (15.4)
Specific phobia	3 (11.5)
Social phobia	1 (3.8)
OCD	1 (3.8)

*OCD*  obsessive–compulsive disorder

### Posttraumatic stress

At intake, PTSD severity was high according to both youths and caregivers. Participants’ PTSS decreased significantly from T1 to T2, *F*(1, 18) = 11.41, *p *= .003, according to the CATS in self-report. The symptom reduction was significant for the completer sample at both T3, *F*(1, 16) = 10.49, *p *= .005, and T4, *F*(1, 13) = 12.63, *p *= .004. Within group effect sizes (Cohen’s *d*) were high in all comparisons (Table 2). With regard to proxy report, PTSD overall symptoms showed a significant decrease at T2, *F*(1, 18) = 90.01, *p *< .001, and consequently high effect sizes (Table 2). This was evident for T3, *F*(1, 16) = 94.73, *p *< .001, and T4, *F*(1, 13) = 33.04, *p *< .001. Reliable change according to the RCI was achieved in 37.4% (n = 9) of cases according to self-report. Caseness (Kinder-DIPS) fell significantly from 100% at T1 to 16% at T2, a recovery rate of 84% (n = 16).

**Table 2:** PTSD symptoms and effect sizes at baseline and post-intervention, 6-weeks and 6-months follow-up

	T1–T2 (n = 19)			T1–T3 (n = 17)			T1–T4 (n = 14)		
	M_1_ (SD)	M_2_ (SD)	d	M_1_ (SD)	M_3_ (SD)	d	M_1_ (SD)	M_4_ (SD)	d
CATS self	30.58 (7.16)	20.16 (11.63)	1.08	30.94 (7.40)	20.35 (11.34)	1.11	30.50 (6.56)	17.86 (12.94)	1.23
CATS proxy	33.16 (5.72)	17.53 (7.24)	2.40	33.65 (5.77)	17.06 (5.45)	2.95	32.50 (5.57)	17.00 (7.33)	2.38

*T1* baseline, *T2* post-intervention, *T3* 6-weeks follow-up, *T4* 6-months follow-up, *CATS* Child and Adolescent Trauma Screen

### Secondary outcomes

The repeated measures MANOVA revealed a significant effect for caregiver-reported comorbid depressive symptoms, *F*(2, 18) = 15.84, *p *< .001. We observed a significant symptom reduction at T2 and T3, and high effect sizes for the post hoc comparisons (see Table 3). The same picture emerged for caregiver-reported behaviour problems with a significant effect in the MANOVA, *F*(2, 18) = 8.90, *p *= .002, and significant post hoc t-tests. As physical complaints showed a significant effect, *F*(2, 18) = 4.15, *p *= .033, we computed post hoc t-tests for T1–T2 and T1–T3 comparisons. A significant decrease in symptoms was observed at T2 only. There was a significant effect for self-reported behaviour problems, *F*(2, 18) = 4.07, *p *= .035. Post-hoc t-tests yielded a trend towards a significant symptom reduction at T2 only, as shown in Table 3. Self-reported depressive symptoms showed no significant mean effect in the MANOVA, *F*(2, 18) = 1.48, *p *= .255. However, participants had already scored below the cut-off at baseline.

**Table 3:** Post-hoc t-tests and effect sizes for symptom changes from T1 to T2 and T1 to T3 for secondary outcomes: depressive, behaviour and physical symptoms

	T1–T2					T1–T3				
	n	M_1_ (SD)	M_2_ (SD)	t	d	n	M_1_ (SD)	M_3_ (SD)	t	d
MFQ proxy	19	13.32 (4.26)	5.63 (4.52)	8.52***	1.75	18	13.50 (4.30)	6.17 (4.89)	5.56***	1.59
SDQ self	18	13.72 (5.33)	10.28 (6.54)	1.86^†^	0.58	17	14.00 (5.36)	11.76 (7.26)	1.05	0.35
SDQ proxy	18	16.67 (5.24)	9.33 (5.17)	7.26***	1.41	17	17.00 (5.20)	9.94 (5.87)	5.06***	1.27
PHQ-15	16	9.06 (3.68)	6.56 (4.24)	2.60*	0.63	15	8.87 (3.72)	7.53 (5.83)	0.97	0.27

*T1* baseline, *T2* post-intervention, *T3* 6-weeks follow-up, *MFQ* Mood and Feelings Questionnaire, *SDQ* Strengths and Difficulties Questionnaire, *PHQ-15* Patient Health Questionnaire Physical Symptoms;

^†^*p *< .1; *p < .05; ***p < .001

### 6-month follow-up and asylum procedures

**Fig. 2:**
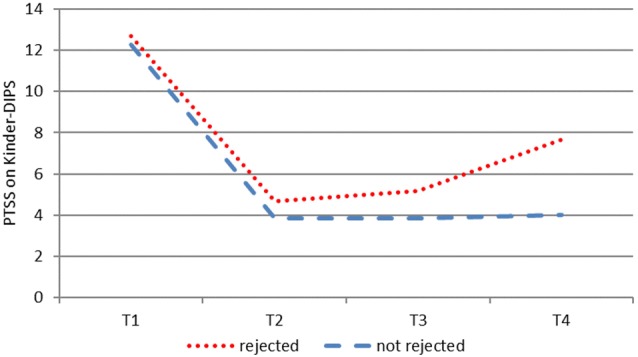
Course of PTSD symptoms (Kinder-DIPS) of completers at T4 (n = 15). Sub-sample with rejected asylum request n = 8 and without rejected asylum request n = 7

**Fig. 3:**
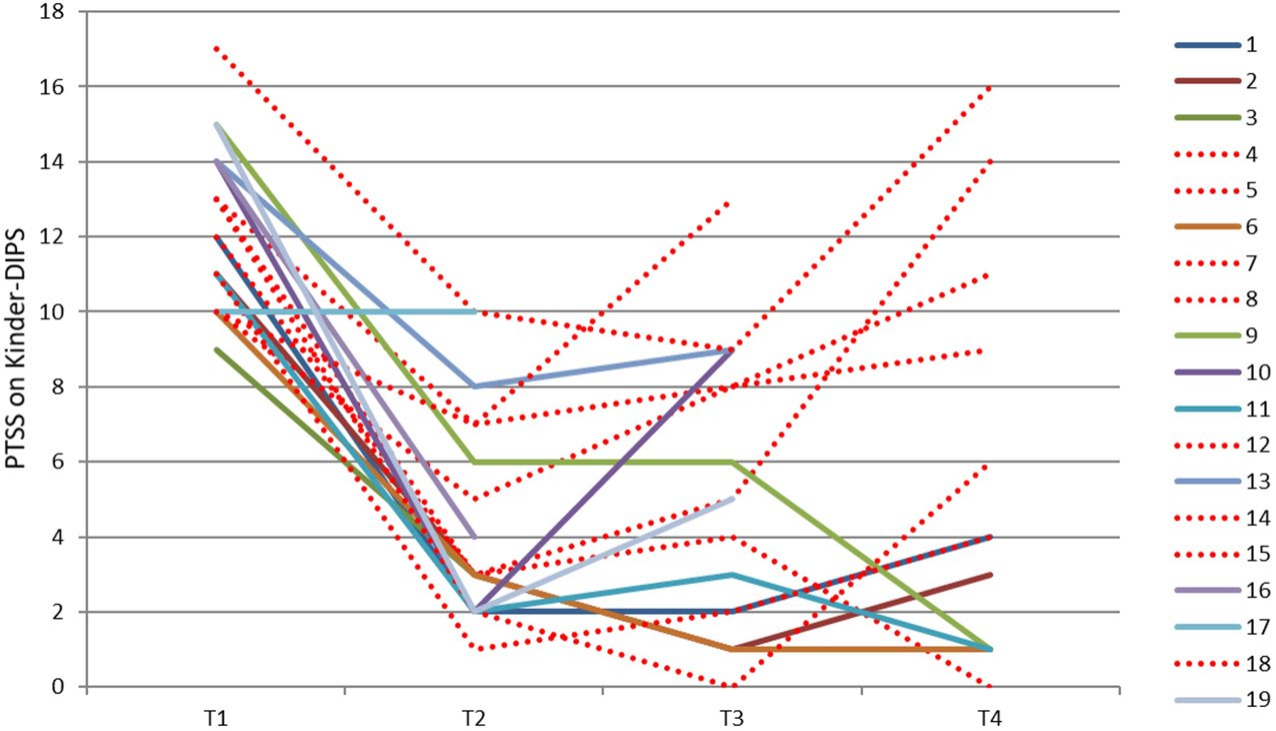
Individual trajectories of PTSD symptoms (Kinder-DIPS) of completers (n = 19). Dotted lines indicate URMs with rejected asylum request

To explore the effects of asylum status, we present an illustration of trajectories of completers in Figs. 2, 3 (PTSD symptoms according to Kinder-DIPS interview). We divided the T4 sample into two sub-samples: rejected asylum request and no rejected asylum request (i.e. waiting for asylum hearing, waiting for asylum decision, or asylum granted). Based on the visual inspection we analysed the two groups for differences in PTSD symptoms. While Fig. 2 suggests that those who did not receive an asylum rejection maintained their improvements at T4 and those with a refusal showed an increased number of symptoms, the statistical analysis did not yield a significant difference between these two groups. On the individual level, however, the illustration (Fig. 3) suggests that those who had a rejected asylum request after the end of therapy (red dotted lines) frequently deteriorated. These conclusions are drawn from the illustrations only and are separate from the statistics.

## Discussion

We report on the efficacy of individual outpatient TF-CBT for URMs in an uncontrolled pre-post design with two follow-up assessments. PTSD levels were high at intake and the sample presented as highly distressed in terms of traumatic events, the number of losses and suicidal and self-harming behaviour in the past. We observed a statistically significant improvement in PTSD symptoms on the group level and a significant reduction in PTSD cases at post-treatment. These findings were supported by large effect sizes and were evident for PTSD symptoms at both T3 and T4. Depressive symptoms and behaviour problems decreased significantly according to the caregiver report and remained stable at the follow-ups. The participants’ physical health problems improved significantly after treatment. Charting individual trajectories revealed that some participants’ PTSD symptoms deteriorated 6 months after the end of therapy. We found some pointers that the rejection of asylum has the potential to increase PTSD-related distress in URMs who had initially benefited.

Our sample characteristics support previous findings that URMs constitute a severely distressed group of patients regarding PTSD, depression and suicidality [5, 9]. This is the first systematic trial on individual PTSD psychotherapy in URMs. Effect sizes were slightly higher than in a pilot study for a group prevention using a TF-CBT like approach for URMs [37] and were comparable to pilot trials for individual therapy with non-refugee adolescents [38]. We observed a significant symptom reduction and high effect sizes despite diverse cultural backgrounds and the involvement of interpreters. Consequently, this underlines that TF-CBT is a robust and culture sensitive intervention [18]. It can contribute to improved mental health care for the population of URMs.

We found several factors in this study that support the feasibility of TF-CBT as an evidence-based treatment for this population. Treatment fidelity checks enabled us to investigate whether TF-CBT was conducted by the therapists as indicated. While this was the case for sessions with the participants, treatment fidelity was only moderate with regard to caregiver sessions. This can be explained by the high age of participants that asked for less caregiver involvement than usual und some modifications (for instance, less focus on parenting skills, more focus on preparation of support for asylum hearing). With only two treatment cases that were terminated by participants during the course of the intervention and two to three cases that were lost to each follow-up assessment, there was a low dropout rate for this type of sample [25]. This indicates that the steps taken to keep participants in treatment were mostly successful. Caregivers played an important role in encouraging participants to stay in treatment. We succeeded in involving a caregiver in all treatment cases. This is a huge achievement, given the difficulties URMs experience with trusting others, the losses they have experienced and the high work load of caregivers in the facilities. PTSS severity at intake and its improvement reported by the caregivers were comparable to the self-report, indicating that they were able to provide a reliable estimation of the participants’ distress. This runs contrary to the findings of Pfeiffer and colleagues [39]. The number of cases that showed a reliable symptom change was rather low. However, the self-report measure was used to analyse this, and we see two possible limitations here. First, self-reported symptom levels at baseline were surprisingly low in comparison to the clinical rating in some cases. Hence, there was not as much room for improvement as expected. And secondly, we have to keep in mind that URMs are a sample with many stressors even after the end of therapy. The severity score of the CATS does not, however, take into account how much the participants were limited in their daily functioning. For instance, while sleep disturbances might still be evident in a participant at T2, he may be less burdened by them in comparison to T1. In addition, we observed high recovery rates in the clinical interview which further support the feasibility of TF-CBT.

In line with previous research [9, 10] we were able to document the distress that was related to the asylum process. In addition to previous findings in URMs who did not receive psychotherapeutic care, our data suggest an impact on youth who had been successfully treated. The mental health of URMs seemed to be destabilized by the anticipation of a repeated confrontation with actual trauma reminders. When we discussed the content of the anticipated catastrophes the participants were afraid of, it became clear that the fear was often realistic and not extreme. The asylum decisions were life or death decisions for many young refugees. Despite circumstances that cannot be judged as safe, risk of suicidality, and a high dosage of traumatic experiences, it is feasible and necessary to provide evidence-based treatments for this target group as supported by the outcomes of our study.

There are some limitations that deserve attention. First, the uncontrolled design and the small sample size for an intervention study limit the strength of the conclusions that can be drawn from the findings. Hence, an RCT with a solid sample size is necessary to test the efficacy of TF-CBT with URMs. Second, some participants were not available for follow-up assessments. This reduced the sample size and posed the question as to how they could have been kept in the study. This, and our inclusion criteria, limit the generalizability of our sample as we only included severely distressed participants with a PTSD diagnosis. Recent research has, however, shown that even moderately distressed URMs can profit from a trauma-focused group intervention [39]. Third, in diagnostic as well as in therapeutic sessions, interpreters assisted with communication which may have led to some loss of information and misunderstandings that we cannot control. Nevertheless, in treatment sessions with translators, participants listened to their trauma narratives in two languages and, therefore, twice as often as usual. Furthermore, translators can support therapists in understanding some cultural characteristics and build a bridge for culturally sensitive therapeutic work. Fourth, we found a Cronbach’s alpha in a satisfactory range for some proxy report measures. Most of these measures assessed internalizing symptoms which are difficult for caregivers to judge. This could be one reason for the moderate reliability. In addition, there was a low level of agreement between the interview and the MFQ regarding depressive symptoms. Last, the sample size at T4 was not large enough to statistically analyse the influence of rejected asylum requests on therapy outcomes, which was solely described with the help of illustrations. Further studies into the influence of political decisions on the mental health of young refugees are needed to underline their need for protection.

## Conclusions

This pilot study demonstrated that obstacles to research with URMs can be overcome. We replicated our initial findings that TF-CBT is feasible and promising for the treatment of URMs with PTSD [20], and we added some important statistical data. An RCT including long-term follow-ups should be the next step in evaluating evidence-based PTSD-treatments for URMs, possibly within a stepped care design to support not only those who have been diagnosed with PTSD but also to bring about a major improvement in mental health care for this population. The involvement of professional caregivers is an important key to the successful treatment of URMs. It not only secures attendance but also helps rebuild the social network that URMs lack. It is important to mention that the refusal of asylum may lead to increased distress in these youths and may constitute a renewed traumatic experience. We need to do more research on this in order to inform policymakers about the vulnerability and need for protection of URMs. Nevertheless, our findings can help to convince psychotherapists that this target group can be treated with an evidence-based treatment even if their life circumstances are not as safe as in other patients.

## Data Availability

The datasets used and analysed during this study are available within reason from the corresponding author.

## Abbreviations

CATS: Child and Adolescent Trauma Screen
CAW: child and adolescent welfare
DSM-5: Diagnostic and Statistical Manual of Mental Disorders, 5th edition
Kinder-DIPS: Diagnostic Interview for Mental Disorders in Childhood and Adolescence
MANOVA: multivariate analysis of variance
MFQ: Mood and Feelings Questionnaire
PHQ-15: Patient Health Questionnaire Physical Symptoms
PTSD: posttraumatic stress disorder
RCI: reliable change index
RCT: randomized controlled trial
SDQ: Strengths and Difficulties Questionnaire
TF-CBT: trauma-focused cognitive behavioural therapy
URMs: unaccompanied refugee minors

## Footnotes

1 To facilitate reading, the term unaccompanied refugee minor will hereafter apply to both unaccompanied asylum seeking and refugee minors.
